# Assessing variant effect predictors and disease mechanisms in intrinsically disordered proteins

**DOI:** 10.1371/journal.pcbi.1013400

**Published:** 2025-08-19

**Authors:** Mohamed Fawzy, Joseph A. Marsh

**Affiliations:** MRC Human Genetics Unit, Institute of Genetics and Cancer, University of Edinburgh, Edinburgh, United Kingdom; Tel Aviv University, ISRAEL

## Abstract

Intrinsically disordered regions (IDRs) are central to diverse cellular processes but present unique challenges for interpreting genetic variants implicated in human disease. Unlike structured protein domains, IDRs lack stable three-dimensional conformations and are often involved in regulation through transient interactions and post-translational modifications. These features can affect both the distribution of pathogenic variants and the performance of computational tools used to predict their effects. Here, we systematically assessed the distribution of pathogenic *vs* benign missense variants across disordered, intermediate, and structured protein regions in the human proteome. Pathogenic variants were notably depleted in IDRs yet were associated with distinct molecular mechanisms, particularly dominant gain- and loss-of-function effects. We evaluated 33 variant effect predictors (VEPs), revealing widespread reductions in sensitivity for pathogenic variants in IDRs, despite high AUROC scores driven by accurate benign variant predictions. We also observed substantial discordance among VEP classifications in disordered regions, underscoring the need for region-aware thresholds and disorder-informed prediction strategies. Incorporating features reflective of IDR biology, such as transient interaction motifs and modification sites, may enhance the accuracy and interpretability of future tools.

## Author summary

Some parts of proteins, known as intrinsically disordered regions, do not fold into fixed shapes. Instead, they stay flexible and play key roles in controlling how cells work, often by forming short-lived interactions or responding to signals. Because of this flexibility, these regions are harder to study, especially when it comes to understanding how genetic changes might lead to disease. In this study, we looked at how often disease-causing mutations appear in disordered regions, and how well current computational tools can spot them. We found that these tools often struggle to detect harmful mutations in disordered parts of proteins, even though they seem to perform well overall. This is because they are good at identifying harmless variants, which are common in these regions, but tend to miss the rare ones that do cause problems. We also found that different tools often give conflicting answers when looking at the same variant in a disordered region. Our results show that new methods are needed to properly interpret genetic changes in these parts of proteins, taking into account the unique ways they work and fail.

## Introduction

Intrinsically disordered regions (IDRs) of proteins lack stable secondary or tertiary structure under physiological conditions, instead adopting flexible conformations that can shift in response to binding partners or cellular cues [1–3]. This structural plasticity allows IDRs to act as molecular hubs in diverse cellular processes such as transcriptional regulation, signal transduction, and protein–protein interactions [4]. Their interactions are typically transient and reversible, often mediated by short linear motifs, post-translational modification (PTM) sites, or segments involved in phase separation [2,5]. Although some IDRs engage in high-affinity or promiscuous binding, their flexibility enables rapid responses to cellular signals and extensive PTM, allowing fine-tuned regulation in dynamic environments [6–9]. IDRs are especially prevalent in eukaryotic proteins, with around 30–40% containing long disordered regions (>30 residues) [10]. Their sequence composition, enriched in polar and charged residues, and depleted in hydrophobic residues, prevents stable folding, favouring a dynamic equilibrium of conformational states [1,11,12]. These properties make IDRs central to regulatory networks and sensitive to perturbation, consistent with their involvement in a range of human diseases, including cancer, cardiovascular, neurodegenerative, and prion disorders [9].

Variant effect predictors (VEPs) are computational tools designed to estimate the potential impact of genetic variants, particularly on human health and disease. VEPs vary widely in their algorithms, training data, and input features, such as evolutionary conservation, structural properties, allele frequencies, or functional assay data [13]. While VEPs are widely used, relatively little attention has been given to how their performance varies depending on whether a variant is in a structured or disordered region. Despite the functional importance of IDRs, these regions might pose significant challenges for VEPs. Unlike structured protein domains, IDRs tend to be less evolutionarily conserved and lack the fixed secondary and tertiary structures that some VEPs rely on as predictive features. As a result, VEPs may struggle to accurately classify pathogenic variants in these regions. Previous studies have shown that standard predictors such as SIFT and PolyPhen-2 exhibit a reduction of more than 10% in sensitivity for pathogenic variants in IDRs compared to structured regions [14]. However, a systematic evaluation of VEP performance across different protein structural contexts remains largely unexplored.

Previously, we showed that IDRs significantly influence the apparent performance of missense VEPs, often leading to inflated area under the receiver operating characteristic curve (AUROC) values [15]. This elevation in AUROC stems from the fact that disordered regions are enriched with putatively benign missense variants, which are typically under weaker evolutionary constraint and thus easier for VEPs to correctly classify as benign. However, this can be misleading: while it is not technically incorrect, it primarily reflects the ease with which VEPs handle these straightforward benign classifications rather than an ability to detect disease-causing mutations. This distinction is critical, as it suggests that the impressive AUROC values observed in proteins with long disordered regions may not indicate robust performance in identifying pathogenic variants, which is often the primary concern in clinical and research settings. In the extreme case, a predictor that labels every variant in an IDR as benign would achieve a deceptively high AUROC, despite having no real ability to detect pathogenic variants. This highlights the need to evaluate sensitivity and class balance alongside overall accuracy metrics.

Building on this observation, here we have further investigated the occurrence of pathogenic missense variants and the predictive performance of VEPs in IDRs. First, we systematically classified residues across the human proteome to assess the distribution of pathogenic and benign variants in disordered versus structured regions. We evaluated 33 VEPs, spanning clinical-trained, population-tuned, and population-free categories, to determine their effectiveness in identifying pathogenic variants within IDRs. Our analysis reveals significant differences in variant distribution and VEP performance across structural contexts, highlighting the limitations of current predictors in capturing the subtle functional impacts of variants in disordered regions. Furthermore, we explored the molecular mechanisms driving pathogenicity in IDRs, particularly in autosomal dominant genes, and propose strategies to enhance VEP accuracy by incorporating region-specific thresholds and IDR-specific features. These findings provide a foundation for refining computational tools to better interpret the effects of genetic variants in disordered regions, with implications for understanding their role in human disease.

## Results and discussion

### Pathogenic missense variants are depleted in disordered regions

To define intrinsically disordered regions, we used AlphaFold2 (AF2) pLDDT scores, which correlate inversely with structural order [16–18]. AF2 pLDDT is recognised as a robust predictor of disorder [17]. While some studies suggest that pLDDT may approach or even exceed the performance of traditional disorder prediction tools in certain contexts [19,20], benchmarking efforts such as CAID-2 [21] have shown that no single method consistently outperforms others across all benchmarks. Thus, we use pLDDT here primarily due to its consistent proteome-wide availability and demonstrated utility for defining long disordered regions.

**Fig 1:**
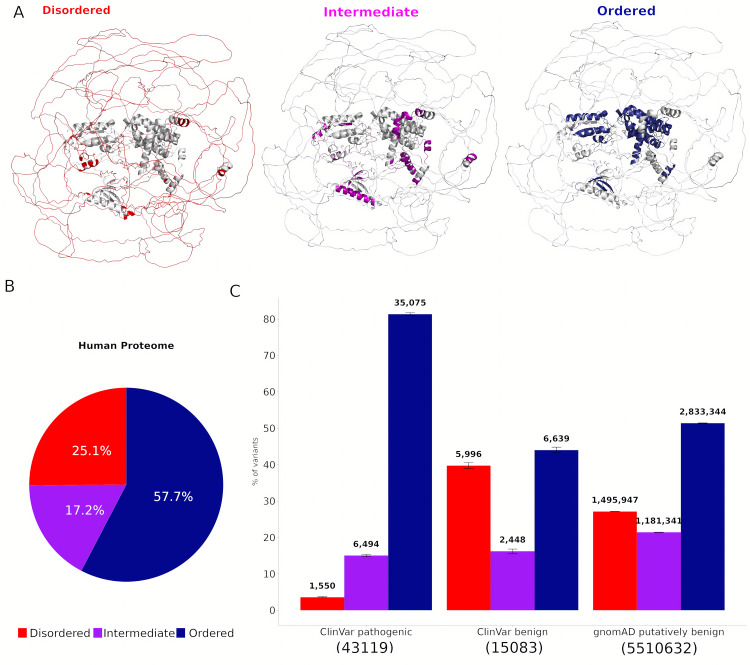
Rarity of pathogenic mutations in intrinsically disordered regions. **(A)** AF2-predicted structure of human Rho GTPase-activating protein 32 (UniProt ID: A7KAX9), with disordered, intermediate, and ordered regions depicted in red, purple, and blue, respectively. **(B)** Proportion of residues classified as disordered, intermediate, and ordered across the human proteome. **(C)** Distribution of missense variants across structural regions, expressed as percentages, with pathogenic and likely pathogenic variants from ClinVar, benign and likely benign variants from ClinVar, and putatively benign population variants from gnomAD.

Residues were classified as disordered, ordered, or intermediate based on pLDDT and local context, as illustrated in Fig 1A (see Methods). Importantly, our approach uses a conservative definition: only residues within long, low-confidence stretches (≥30 residues with average pLDDT <70) were classified as disordered. This threshold aims to capture regions likely to be truly unstructured under physiological conditions, such as extended flexible linkers or regulatory activation domains, rather than transiently mobile loops within structured domains. Shorter, low-confidence segments were instead assigned to an intermediate category to reduce false positives, particularly in cases where local flexibility does not imply global disorder. This strict classification likely underestimates the full extent of disorder in the proteome, but provides a high-confidence set of IDRs for downstream analysis.

Applying this approach to 20,281 canonical human proteins, we found that 25.1% of residues fall within disordered regions (Fig 1B). In addition, 57.7% of residues are predicted to be ordered, while 17.2% are classified as intermediate. When considering disorder at the level of human proteins, most (57.7%) are predicted to have at least one disordered region, according to our definition, while 26.2% are highly disordered, having at least 30% of their residues in disordered regions (Fig A in S1 Text). These observations are broadly consistent with previous findings [18,22], noting our strict disorder definition, as well as our more permissive intermediate classification.

We next examined the distribution of missense variants across structural regions. Variants were grouped into three categories: pathogenic and likely pathogenic variants from ClinVar [23], benign and likely benign variants from ClinVar, and putatively benign population variants from gnomAD [24]. Pathogenic variants were strongly depleted in disordered regions, with only ~3.6% occurring at disordered residues, compared to 15.1% in intermediate and 81.3% in ordered regions (Fig 1C). In contrast, ClinVar benign variants were heavily enriched in disordered regions (39.8%). Finally, 27.2% of gnomAD putatively benign variants occurred in disordered regions, showing a slight enrichment over the occurrence of disorder in the human proteome. Overall, the vast majority of variants found in disordered regions are benign or putatively benign, while only a small minority are classified as pathogenic.

The strong enrichment of pathogenic missense variants in ordered regions and the corresponding enrichment of benign variants in disordered regions is broadly consistent with previous findings [25–27]. This distribution reflects fundamental differences in structural constraints between these regions. In a disordered region, a single amino acid substitution is unlikely to cause major functional disruption unless it affects a specific critical residue, such as one involved in PTMs or transient binding interactions. Conversely, in a structured domain, even a single missense variant can have a profound effect by destabilising the fold, altering interaction surfaces, or disrupting active sites. This difference in mutational tolerance aligns with the expectation that proteins rely on stable structural elements for core functions, whereas disordered regions often accommodate greater sequence variation without loss or change of function. Nevertheless, despite their depletion, we identified 1550 pathogenic missense variants in disordered regions, demonstrating that such variants can and do contribute to disease.

### Distinct mechanisms underlie pathogenic variants in disordered regions

Pathogenic missense variants in disordered regions must operate through distinct molecular mechanisms compared to those in ordered regions. In globular domains, such variants often cause disease by destabilising protein structure, leading to misfolding, or affecting the active site and ultimately a complete loss of function. In contrast, in disordered regions, pathogenic variants may affect protein behaviour by subtly altering regulatory interactions or disrupting specific binding motifs, such as short linear motifs or PTMs, without necessarily abolishing the protein’s overall function [28]. Beyond direct loss of interactions, recent work has shown that mutations in IDRs can perturb the conformational ensemble of the protein. Rather than adopting a fixed structure, IDRs exist as dynamic ensembles of interconverting conformers, and single-point mutations can shift this ensemble in ways that affects function, binding preferences, or regulatory activity [29]. These effects may be indirect but are functionally significant, especially in regulatory contexts.

In addition, LLPS is now recognized as a key mechanism by which many disordered regions, particularly in transcription factors and RNA-binding proteins, facilitate the formation of membraneless biomolecular condensates [30]. These condensates play critical roles in organizing cellular biochemistry, concentrating macromolecules at specific genomic loci, and regulating gene expression. Pathogenic variants in activation domains or other phase-separating IDRs can alter the properties or assembly behaviour of these condensates, impeding their formation or modifying their composition and dynamics. Thus, LLPS disruption represents another important mechanism by which variants in IDRs can cause disease, especially in contexts where dynamic compartmentalisation and multivalent interactions are essential for normal function. Complementing these observations, recent proteome-wide simulations showed that pathogenic missense variants in IDRs are disproportionately located in regions with low conformational entropy [31]. These findings support the notion that even subtle shifts in the structural ensemble of IDRs can be pathogenic, especially when they occur at dynamically constrained regulatory segments.

**Fig 2:**
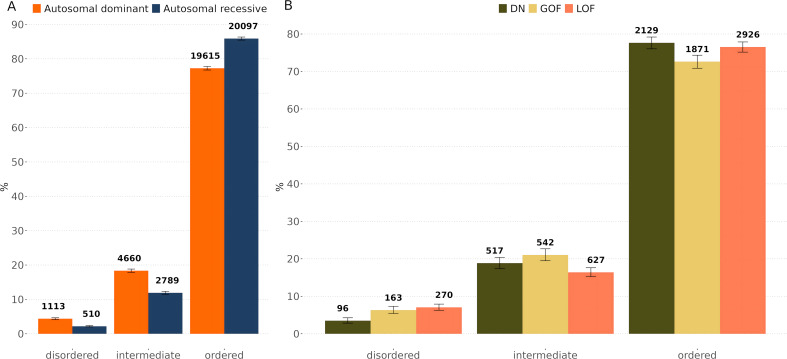
Distribution and counts of pathogenic missense variants by inheritance mode and molecular mechanism. **(A)** Bar plot illustrating the proportions and counts of pathogenic missense variants across disordered, intermediate, and ordered structural regions in autosomal dominant (AD) and autosomal recessive (AR) genes. Counts of pathogenic variants per structural region and inheritance mode are indicated above each bar. **(B)** Bar plot showing the percentage and count of pathogenic missense variants associated with dominant negative (DN), gain-of-function (GOF), and loss-of-function (LOF) mechanisms within AD genes across structural regions. Counts are displayed above each bar, with error bars representing 95% binomial confidence intervals.

Given that current VEPs tend to perform best on loss-of-function (LOF) variants compared to gain-of-function (GOF) and dominant-negative (DN) variants [32], we can speculate that they might perform less well at the identification of pathogenic variants in disordered regions. To explore this further, we first examined inheritance mode as a proxy for mechanism. Variants in autosomal recessive (AR) disorders nearly always act via LOF, while those in autosomal dominant (AD) disorders can involve LOF, GOF, or DN effects [33,34]. In Fig 2A, we plot the proportion of variants in AD *vs* AR genes that occur in disordered, intermediate and ordered regions. Although the large majority of pathogenic variants are in ordered regions for both categories, it is interesting to note that AD genes show roughly double the proportion of pathogenic variants in disordered regions compared to AR genes (4.4% *vs* 2.2%, P = 3 x 10^-137^, Fisher’s exact test). Similarly, pathogenic variants in AD genes are also moderately enriched in intermediate regions (18.4% *vs* 11.9%, P = 1 x 10^-87^, Fisher’s exact test). This suggests that pathogenic variants in AR genes are more likely to act via a LOF mechanism involving structural disruption in an ordered region. In contrast, a much greater proportion of pathogenic variation in AD genes occurs in disordered regions, likely acting via more complex mechanisms related to the role of disordered regions in regulation, signalling, and interaction networks.

Next, we considered pathogenic variants from AD genes, and grouped them by molecular mechanism (LOF, GOF and DN) based on a previously published classification [32] (Fig 2B). Surprisingly, LOF variants showed the highest enrichment in disordered regions, in marked contrast with our initial expectation based on the results of the AR *vs* AD variants, and our naïve expectation of how pathogenic variants in disordered regions would be likely to act. Interestingly, however, LOF variants showed the lowest representation in intermediate regions. On closer consideration, we noted that many of the disordered LOF variants in AD genes occurred in transcription factors, which have a very well-known association with haploinsufficiency [35]. Given that many transcription factors contain long disordered regions, often playing important roles in transactivation [30,36], this suggests that disruption of these functions is a common mechanism for pathogenicity of variants in disordered regions.

GOF variants were also enriched in disorder compared to AD variants in general, with 6.3% occurring at predicted disordered regions. Interestingly, GOF variants also showed the strongest enrichment at intermediate regions, and the lowest representation in ordered regions. This is consistent with the previous observation that GOF variants were enriched in regions with lower pLDDT values [32]. This pattern is consistent with the flexible, context-dependent nature of disordered and intermediate regions, which often mediate transient, regulated interactions. GOF variants in these regions may create new or stronger interactions, disrupt phase separation, interfere with PTM sites, or perturb short linear motifs involved in dynamic regulation. These changes can enhance or misdirect protein activity without necessarily compromising stability or folding, making disordered and intermediate regions plausible sites for such effects.

Several well-known GOF mutations illustrate these principles. The canonical oncogenic BRAF p.Val600Glu mutation [37,38] occurs in a region predicted to be disordered in our pipeline and activates the MAPK pathway by mimicking phosphorylation. The histone variant H3F3A p.Lys27Met [39], found in paediatric gliomas, disrupts PRC2 binding at the disordered N-terminal tail, altering the epigenetic landscape. In FOXL2, a transcription factor associated with adult granulosa cell tumours, the p.Cys134Trp variant [40] lies within a disordered region flanking the DNA-binding domain and is thought to alter gene expression via changes in interaction specificity or post-translational control. These cases illustrate how disordered regions can host GOF mutations that bypass normal regulation without affecting global protein structure.

In contrast, only 2.2% of DN variants occurred at disordered regions, much lower than for the other AD mechanisms. This is consistent with the DN mechanism’s strong association with oligomeric interfaces, which tend to occur in structured domains [32]. While rare, DN effects in disordered regions remain plausible in cases involving misregulation of binding motifs or competitive inhibition [33].

### VEP performance varies across structural regions

Next, we investigated how VEPs perform in distinguishing pathogenic from putatively benign missense variants across disordered, intermediate and ordered regions. We used 33 different VEPs, with strict coverage filters, so that all VEPs had predictions for all variants tested (see Methods). Although this limits the number of VEPs we can include, it means that our results will not be influenced any coverage biases of individual methods, given that many VEPs lack full coverage even within individual genes [13].

We grouped VEPs into three categories using a recently introduced classification scheme [ 41,42], based on their potential risk of circularity due to their training [43]. *Clinical-trained* VEPs are supervised models trained directly on human variants with known clinical labels, such as pathogenic and benign annotations. These inherently have the highest risk of circularity. *Population-tuned* VEPs are not trained on clinical labels but have been optimised or calibrated using *human population* data, typically through allele frequency-based scaling or tuning. These tend to have a much lower, but non-zero susceptibility to circularity. Finally, *population-free* VEPs have not been trained or tuned on any human variant data and are therefore immune from circularity concerns. This group includes unsupervised methods, protein language models, and models based on evolutionary conservation from sequence alignments.

**Fig 3:**
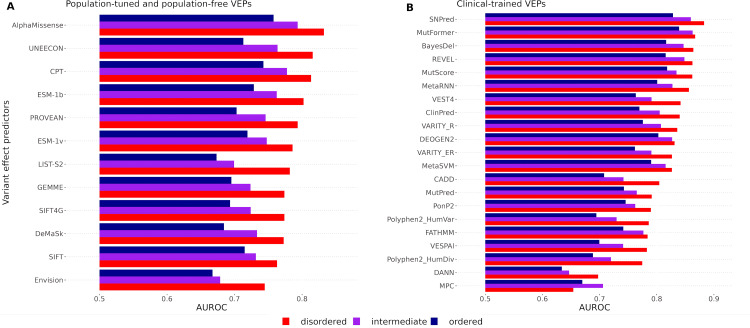
Performance evaluation of VEPs across structural regions. Performance of VEPs assessed by area under the receiver operating characteristic curve (AUROC) across disordered, intermediate, and ordered protein regions. The x-axis represents AUROC values starting at 0.5, and the y-axis lists individual VEPs. Population-free and population-tuned VEPs are shown together in **(A)**, while clinical-trained VEPs are shown in **(B)**.

In Fig 3, we show the AUROC values for disordered, intermediate and ordered regions across all predictors. In Fig 3A, we group population-free and population-tuned VEPs together (noting that most are population-free and only AlphaMissense [44], UNEECON [45] and LIST-S2 [46] are population-tuned). Fig 3B shows the clinical-trained VEPs, which comprise the majority of methods in our analysis. Interestingly, nearly all VEPs show the highest AUROC values in disordered regions and the lowest values in ordered regions. The only exception to this is MPC [47], which shows slightly worse performance in disordered than ordered regions, though we note that its performance across all regions remains poor compared to other VEPs.

When comparing different VEPs, there is little evidence that any methods perform particularly well in specific regions. For example, AlphaMissense and CPT [48] show the highest AUROC of any population-tuned or population-free models across disordered, intermediate and ordered regions. Similarly, SNPred [49] and MutFormer [50] show higher AUROC values than any clinical-trained models across all three regions. Thus, methods that perform well on ordered regions also tend to perform well on disordered regions, and there does not seem to be any reason to recommend a particular VEP for ordered *vs* disordered regions. Notably, VEPs based on protein language models (*e.g.,* ESM-1b [51] and ESM-1v [52]) appear to show very similar trends across regions as those based purely on sequence alignments (*e.g.,* GEMME [53]), suggesting that neither approach has any clear advantages in ordered *vs* disordered regions. This is somewhat surprising, as protein language models have been proposed to capture contextual sequence signals that might help in disordered regions where structural information is lacking [51]. However, our findings suggest that in practice, these models do not currently offer enhanced predictive power in IDRs. This is consistent with a recent benchmark of structure-based predictors, which found that disordered regions substantially reduce predictive performance across multiple architectures and input modalities [54].

### VEPs show low sensitivity for pathogenic variants in disordered regions

The apparently superior performance of VEPs in disordered regions, as measured by AUROC, is consistent with our earlier finding that proteins with large amounts of disorder tend to have higher AUROC values, and that excluding disordered regions often lowers overall performance [15]. In that study, we showed that this effect was not due to improved classification of pathogenic variants, but rather to the high density of putatively benign variants in disordered regions. These sites are typically under weak evolutionary constraint and are more readily classified as benign by most VEPs.

To investigate this phenomenon further, we calculated the sensitivity and specificity at the “optimal threshold” point of ROC curve for each VEP (see Methods). The sensitivity represents the true positive rate: the fraction of pathogenic variants that are correctly classified as pathogenic using this threshold. The specificity represents the true negative rate: the fraction of putatively benign variants correctly classified as non-pathogenic.

**Fig 4:**
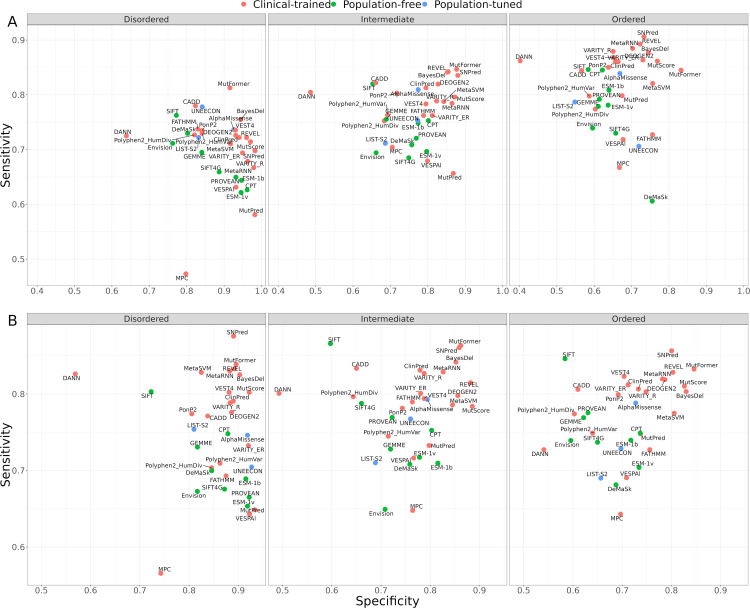
Assessment of VEP performance using global and region-specific optimal thresholds. Performance of VEPs evaluated by sensitivity on the x-axis and specificity on the y-axis. **(A)** Sensitivity *vs* specificity in disordered, intermediate and ordered regions calculated using a global optimal threshold, across all structural regions. **(B)** Sensitivity *vs* specificity calculated using a region-specific optimal threshold.

In Fig 4A, we plot sensitivity *vs* specificity for all VEPs in disordered, intermediate and ordered regions. Strikingly, across nearly all predictors, sensitivity for pathogenic variants in disordered regions was significantly lower compared to ordered and intermediate regions. This low sensitivity indicates that, despite high overall AUROC scores driven by accurate identification of putatively benign variants, VEPs frequently misclassify pathogenic variants within disordered sequences. At the same time, specificities are clearly much higher in disordered regions than ordered regions. That is, while VEPs are less likely to correctly classify variants as pathogenic in disordered regions, they are more likely to misclassify benign variants.

We observe that clinical-trained VEPs tend to have higher sensitivities than population-free VEPs, although this is almost certainly driven by the circularity issue discussed earlier. Interestingly, however, we also note a strong inverse correlation between sensitivity and specificity for population-free VEPs within disordered regions. While some degree of trade-off between these metrics is expected, one plausible explanation is that population-free predictors rely predominantly on evolutionary conservation or biophysical signals that are inherently weaker in disordered regions. As a result, adjusting their decision threshold to capture more pathogenic variants (thus boosting sensitivity) inadvertently increases misclassification of benign variants, reducing specificity. Conversely, a more stringent threshold that accurately dismisses most benign variants sharply lowers sensitivity for the sparse, but functionally impactful, pathogenic variants in IDRs.

Given that the low sensitivity of VEPs in disordered regions means that they are likely to miss true pathogenic variants, we wondered whether the use of region-specific thresholds could be beneficial. Thus, we calculated the optimal thresholds for each VEP from the ROC curves in the same manner as before, but considering only disordered, intermediate or ordered variants (S1 Table). Essentially, this results in lower thresholds for classifying variants as pathogenic in disordered regions, and higher thresholds in ordered regions (assuming a VEP where the score positively correlates with likelihood of pathogenicity). For example, for CPT, the top-performing population-free VEP, we calculate a global optimal threshold of 0.35, compared to a disordered-specific threshold of 0.24 and ordered-specific threshold of 0.44. Note that these are not meant to be thresholds for making clinical classifications; far stricter thresholds would be required for this [55]. Instead, these represent the optimal thresholds for discriminating between pathogenic and putatively benign in our dataset, and therefore suggest useful thresholds for consideration in variant prioritisation.

In Fig 4B, we plot sensitivity *vs* specificity using these region-specific thresholds, while Fig C in S1 Text shows the difference in sensitivity and specificity using region-specific vs global thresholds. The use of region-specific thresholds results in very similar sensitivities of VEPs across all three regions, generally increasing sensitivity in disordered regions and decreasing it in ordered regions. Interestingly, while specificity is also affected, the impact appears to be relatively smaller than on sensitivity in disordered regions. This suggests that the sensitivity gain in disordered regions is likely worth the relatively small impact on specificity. In contrast, the loss of sensitivity in ordered regions is not compensated for by a large specificity increase. Thus, we suggest that these region-specific thresholds are likely useful for disordered and possibly intermediate regions, but they may be of less benefit for prioritising variants in ordered regions.

Given that evolutionary conservation plays a key role in most VEPs, we wondered whether the reduced sensitivity of VEPs could be related to reduced conservation of pathogenic variants in disordered regions. To address this, we compare the residue-level conservation of the sites of pathogenic and putatively benign variants in disordered, intermediate and ordered regions (Fig B in S1 Text). Unsurprisingly, pathogenic variants occur at far more conserved positions than putatively benign variants. Interestingly, however, both pathogenic and putatively benign variants in disordered regions are less conserved residues than those in ordered regions. Thus, the weaker conservation of pathogenic variants in disordered regions means they are less likely to be correctly classified, thus reducing sensitivity, while the weaker conservation of putatively benign variants means that they are more likely to be correctly classified, thus increasing specificity.

Our findings align closely with a recent benchmarking study by Luppino et al. [56], which demonstrated that deep learning-based VEPs such as AlphaMissense maintain high specificity but suffer from markedly reduced sensitivity in IDRs. This elevated false-negative rate reflects the challenge of detecting subtle functional disruptions in regions with weak evolutionary constraints, reinforcing our conclusion that current VEPs are poorly equipped to capture pathogenic effects in disordered contexts.

### VEPs show discordant predictions in disordered regions

Given the observed variability in VEP performance across structural contexts, we next investigated the consistency of their predictions for individual variants. Specifically, we asked whether VEPs tend to agree on which variants are pathogenic in intrinsically disordered regions (IDRs), compared to intermediate and ordered regions.

To quantify agreement, we calculated the average pairwise concordance between VEPs using Cohen’s kappa statistic. This metric quantifies inter-rater reliability while correcting for agreement expected by chance. A kappa value of 1 indicates perfect agreement, 0 corresponds to agreement no better than random, and negative values reflect systematic disagreement. This allows us to assess not just whether two predictors classify variants similarly, but whether they do so more consistently than would be expected by chance alone. We converted VEP scores to binary predictions using each method’s global optimal threshold, and calculated mean pairwise kappa values within and between VEP groups defined by training strategy: clinical-trained, population-tuned, and population-free.

**Fig 5:**
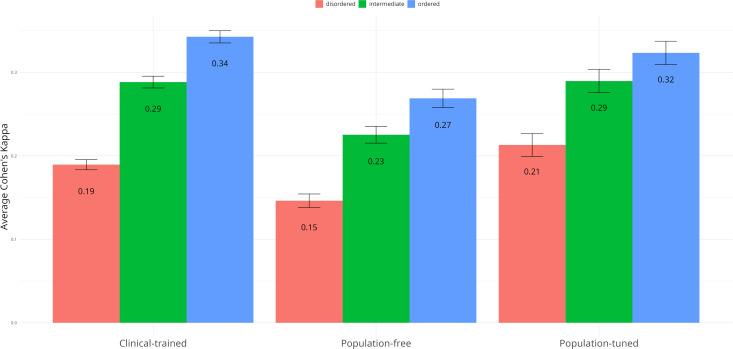
Average classification agreement among VEP groups across structural regions. Average Cohen’s kappa values representing classification agreement among VEP groups across disordered, intermediate, and ordered protein structural regions. The x-axis lists VEP groups categorised by training methodology, and the y-axis indicates the mean Cohen’s kappa for each group within each structural region. Error bars denote the standard error of the mean.

As shown in Fig 5, overall agreement was lowest in disordered regions, particularly among population-free VEPs. These models, which rely primarily on evolutionary conservation and sequence-derived features, appear to disagree more frequently in regions lacking strong structural or conservation signals. This suggests that the sparse and subtle functional constraints characteristic of IDRs lead to reduced model convergence and greater uncertainty in prediction.

In contrast, clinical-trained and population-tuned VEPs showed somewhat higher agreement in disordered regions. This likely reflects shared biases arising from human variant data used during model development or calibration, which can lead to convergence due to circularity rather than true predictive power. Nonetheless, even within these groups, agreement was lower in disordered regions than in ordered regions.

By comparison, all VEP groups showed higher concordance in intermediate and ordered regions, consistent with the stronger evolutionary and structural constraints in these contexts. Here, models are more likely to converge on consistent classifications, likely driven by clearer functional signals.

Together, these findings suggest that disordered regions not only pose challenges for sensitivity but also reduce agreement between VEPs, particularly those without access to human variant data. This highlights the need for specialised strategies, both in training and interpretation, to improve consistency and reliability in these structurally flexible regions.

## Conclusion

IDRs are crucial mediators of diverse regulatory functions, yet their inherent structural flexibility poses significant challenges for accurate interpretation of genetic variants associated with human disease. Our systematic analysis highlights fundamental limitations in current VEPs, which demonstrate notably reduced sensitivity for pathogenic variants within these disordered contexts. Although VEPs consistently achieve higher AUROC scores in IDRs, this largely reflects their proficiency at identifying benign variants, which is driven by weaker evolutionary constraints, rather than their ability to pinpoint subtle yet functionally critical pathogenic alterations. Consequently, relying solely on global thresholds risks overlooking clinically relevant variants that operate through mechanisms unique to disordered regions.

We demonstrate that pathogenic variants in IDRs predominantly act through distinct molecular mechanisms compared to those in structured domains, particularly involving nuanced alterations in regulatory interactions or transcriptional activity. These mechanisms are often inadequately captured by current predictors, especially those relying heavily on evolutionary conservation signals or structured-domain assumptions. Our findings strongly advocate for incorporating region-specific thresholds into VEPs to enhance their sensitivity and accuracy for disordered regions without substantially compromising specificity.

Furthermore, the notable discordance among predictors in classifying IDR variants underscores the importance of refining computational methodologies and training strategies tailored specifically for disordered protein regions. Future VEP development could potentially achieve improved performance and reliability by incorporating region-specific features such as transient binding motifs, PTM sites, and context-dependent structural ensembles. Recent large-scale simulations of human IDRs demonstrate that pathogenic variants are enriched in regions with low conformational entropy, providing a new structural dimension for interpreting variant effects in disordered regions [31]. In parallel, a recent proteome-wide analysis by Cagiada et al. [57] showed that pathogenic variants in IDRs more often disrupt function directly rather than stability, and that these variants are more difficult to model accurately, leading to higher false negative rates with current prediction approaches. This supports our conclusion that disordered regions involve distinct mechanisms of pathogenicity that are poorly captured by models trained predominantly on structured proteins.

Overall, this work provides critical insights into the complexities of interpreting genetic variation within intrinsically disordered regions. It establishes a foundational understanding for future development of more sophisticated computational tools, ultimately enhancing the accuracy of genetic variant interpretation in clinical settings and deepening our understanding of how protein disorder contributes to disease.

## Methods

### Structural classification of human residues

Every residue in the human proteome, considering the primary UniProt isoform of each protein-coding gene was given a structural classification of ordered, intermediate or disordered. To do this, we utilised the pLDDT derived from AF2 [16]. pLDDT inversely correlates very well with the flexibility of protein structures such that AF2 assigns low pLDDT scores for regions that are highly flexible and lack fixed 3D structures such as IDRs and linkers between ordered regions [17,18]. A residue is deemed to belong to a disordered region if its pLDDT is less than 50 and it is part of a contiguous stretch of at least 30 residues with an average pLDDT value less than 70. In contrast, a residue is classified as ordered if its pLDDT is at least 70. Finally, residues falling outside these two conditions were classified as intermediate.

### Missense variant dataset

Our dataset of missense variants was derived in essentially the same way as in our previous study [15], with pathogenic and likely pathogenic variants taken from ClinVar (August 2022) [23], and putatively benign population variants from gnomAD v2.1 [24]. Using gnomAD as a source of ‘putatively benign’ variants is preferable to clinically classified benign variants because it reduces circularity: many VEPs incorporate allele frequency information, which is often used to label clinical benign variants [58], creating a risk of inflated performance due to overlap between training and evaluation data. In contrast, using mostly rare gnomAD variants better reflects the real-world challenge of distinguishing rare benign from rare pathogenic variants while offering a larger, less biased negative class [41,59]. However, we also separately considered benign variants from ClinVar in Fig 1. Importantly, we also removed all missense variants from collagen proteins, by excluding those 213 human protein-coding genes containing collagen-helices, as defined by Pfam (PF01391) [60]. It has been demonstrated previously that presence of collagen-helix containing proteins skew analyses of intrinsic disorder, since they are fibrous proteins but are consistently predicted to be disordered [61]. In our initial analysis, we found that nearly half of pathogenic missense variants in “disordered” regions according to our definition were from collagen proteins. As we do not consider collagens to be intrinsically disordered proteins, we excluded them from our study, and we strongly suggest that people take this into consideration in future studies of disease variants in disordered regions, as they have a high potential to cause confounding.

### Assessing VEP performance and agreement

We started with the set of VEPs tested in a recent benchmarking study (considering those used in the original preprint) [41]. We only retained those methods with scores available for at least 75% of missense variants present in our pathogenic and putatively benign datasets. To ensure consistency of comparisons, we only retained variants with scores shared across all VEPs.

For each VEP, we assessed its performance at distinguishing between pathogenic and putatively benign variants by calculating the AUROC value across different structural regions. We also calculated “optimal thresholds” for distinguishing between pathogenic and putatively benign variants, either on a global basis, or considering specific structural regions. To do this, we applied the Youden J-statistic [62] to the ROC curve and selected the threshold that maximised this value [62]. Using these thresholds, we could then assess whether each VEP predicted each variant to be pathogenic or benign in a binary manner. From this, we could calculate other metrics, including sensitivity and specificity. We also used this to calculate Cohen’s kappa, to assess the level of agreement in classification between each pair of VEPs.

## Supporting information

S1 Text Supplemental figures. File containing additional figures complementary to the analysis shown in the main text of this manuscript.
(DOCX)

S1 Table Global and region-specific optimal thresholds across all VEPs. (DOCX)
