# Supplementary material for: Assessing variant effect predictors and disease mechanisms in intrinsically disordered proteins
Source: PLoS Comput Biol. 2025 Aug 19;21(8):e1013400. doi: 10.1371/journal.pcbi.1013400 (PMC12377588; doi:10.1371/journal.pcbi.1013400)
Supplement: S1 Text — File containing additional figures complementary to the analysis shown in the main text of this manuscript. (DOCX) [file pcbi.1013400.s001.docx]

**S1 Text.**


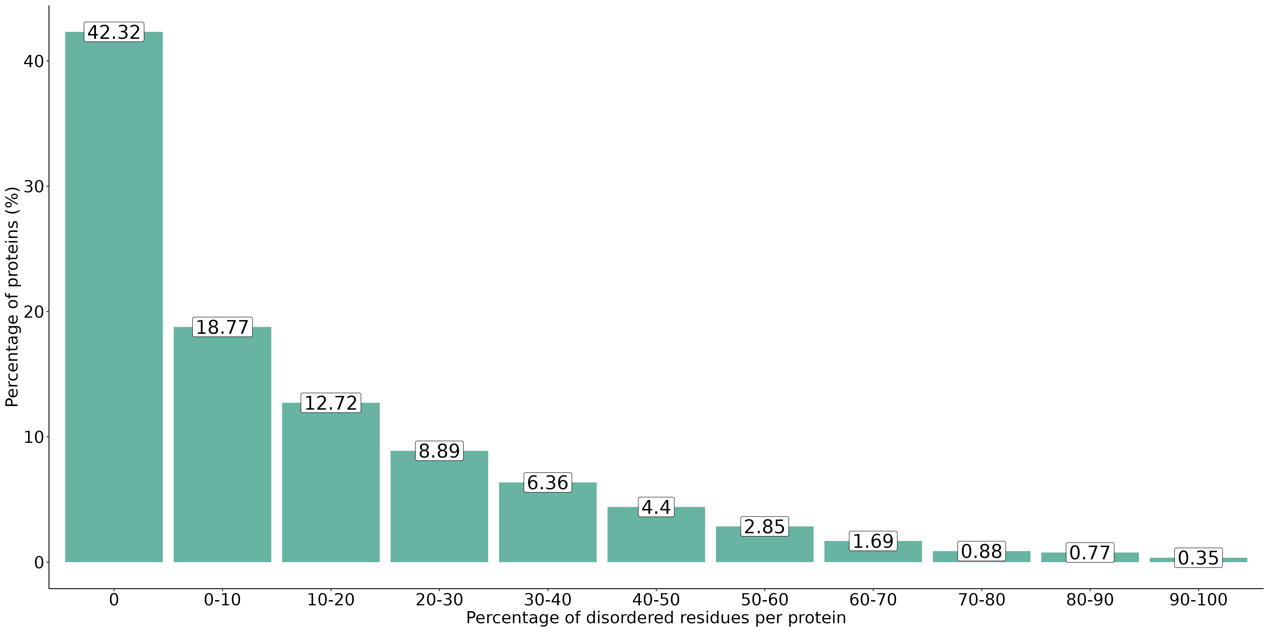


**Fig A. Distribution of human proteins by percentage of intrinsic disorder.** Proportion of human proteins across varying levels of IDRs. The x-axis represents the percentage of IDRs, grouped into bins, and the y-axis shows the percentage of human proteins within each bin. Bins are left-inclusive and right-exclusive (*e.g*., 10.5% falls in the “10–20” bin), while the “0%” bin includes only fully ordered proteins.


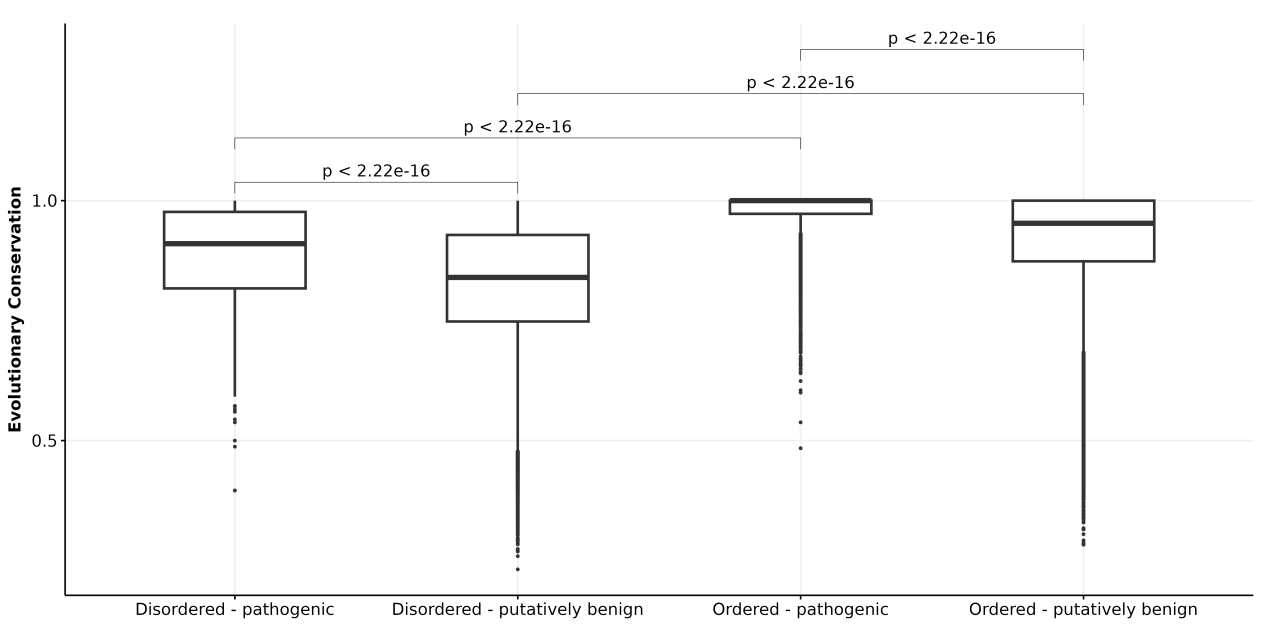


**Fig B. Evolutionary conservation of variants in disordered and ordered regions:** Comparison of evolutionary conservation between pathogenic and putatively benign variants in disordered and ordered protein regions. The x-axis denotes the functional consequence of variants (pathogenic or putatively benign) within each structural region. The y-axis represents evolutionary conservation, calculated as the complement of the relative residue substitution rate (1 - substitution rate) from multiple sequence alignments of closely related homologues using Aminode, with higher values indicating greater residue conservation. P-values, calculated using the Wilcoxon rank-sum test, are displayed for comparisons between groups as indicated.


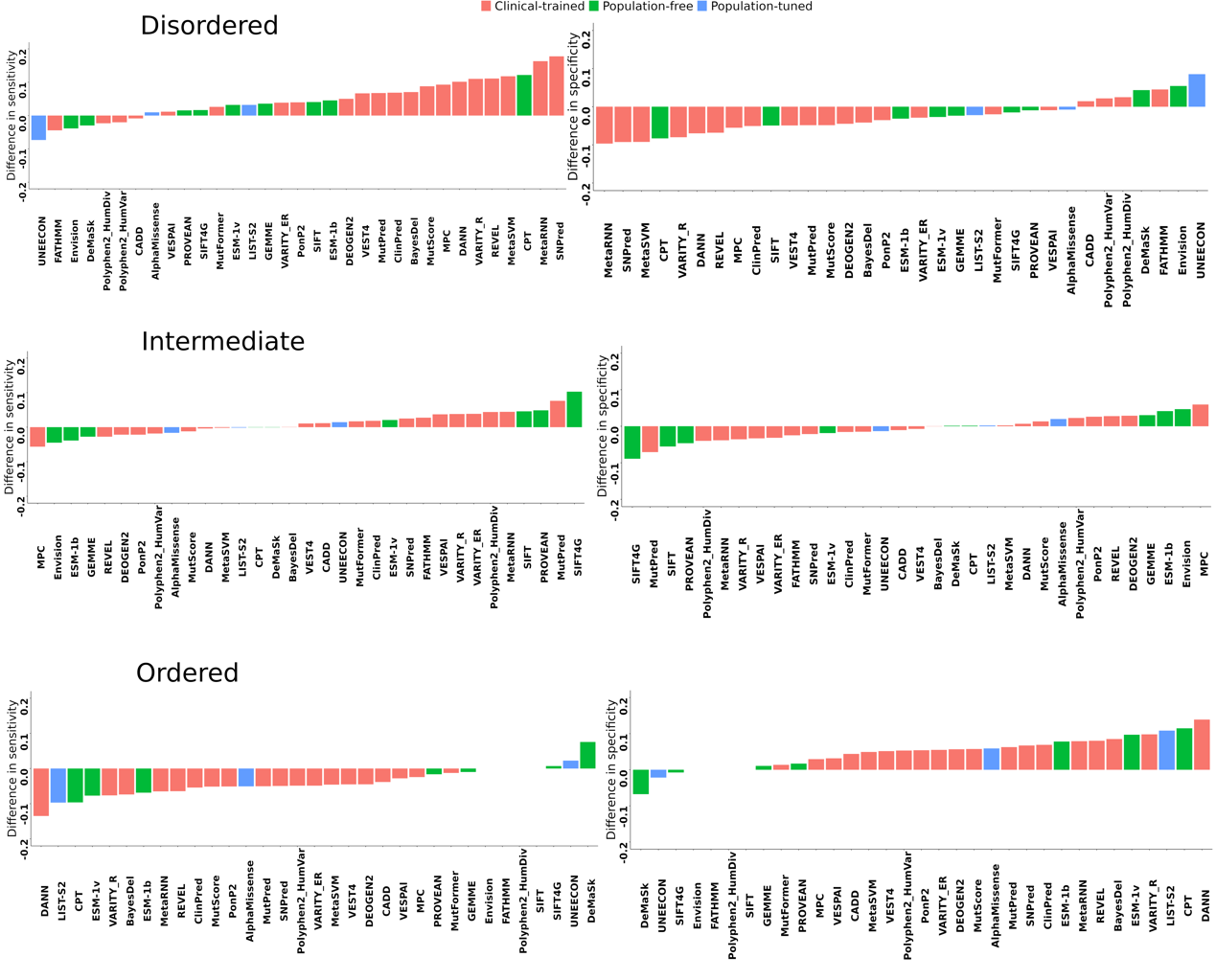


**Fig C. Difference in sensitivity and specificity of VEPs using region-specific *vs* global optimal thresholds.** A sensitivity or specificity difference > 0 indicates that employing the region-specific optimal threshold results in higher sensitivity or specificity the global optimal threshold.
