# Supplementary material for: Assessing variant effect predictors and disease mechanisms in intrinsically disordered proteins
Source: PLoS Comput Biol. 2025 Aug 19;21(8):e1013400. doi: 10.1371/journal.pcbi.1013400 (PMC12377588; doi:10.1371/journal.pcbi.1013400)
Supplement: S1 Table — (DOCX) [file pcbi.1013400.s002.docx]

**S1 Table. Global and region-specific optimal thresholds across all VEPs.**
